# Single incision device (TVT Secur) versus retropubic tension-free vaginal tape device (TVT) for the management of stress urinary incontinence in women: a randomized clinical trial

**DOI:** 10.1186/1756-0500-7-941

**Published:** 2014-12-22

**Authors:** Sue Ross, Selphee Tang, Jane Schulz, Magnus Murphy, Jose Goncalves, Stephen Kaye, Lorel Dederer, Magali Robert

**Affiliations:** Department of Obstetrics and Gynecology, University of Calgary, Calgary, Alberta Canada; Department of Obstetrics and Gynecology, University of Alberta, Edmonton, Alberta Canada; Department of Obstetrics and Gynecology, Penticton Regional Hospital, Penticton, British Columbia Canada; Department of Obstetrics and Gynecology, Lions Gate Hospital, North Vancouver, British Columbia Canada; Cavarzan Chair of Mature Women’s Health Research and Innovation, Department of Obstetrics and Gynecology, Lois Hole Hospital, Royal Alexandra Hospital, University of Alberta, Edmonton, Alberta Canada

**Keywords:** Effectiveness, Randomized controlled trial, Single incision device, Stress urinary incontinence, Surgical treatment

## Abstract

**Background:**

In 2006, Ethicon Inc. introduced a new minimally invasive single incision sling device for the surgical treatment of stress urinary incontinence, the Gynecare TVT Secur®. For device licensing, no new evidence of TVT Secur efficacy and safety was needed: rather evidence was provided of the long-term follow-up of patients who had a procedure using a predecate retropubic tension-free vaginal tape device. Before adopting TVT Secur into our routine clinical practice, we decided to evaluate it. The objective of our Canadian multi-centre pragmatic randomized controlled trial was to compare the effectiveness of the new single-incision device, TVT Secur, to the established TVT device, in terms of objective cure of stress urinary incontinence (SUI) at 12 months postoperatively. Other outcomes included: complications, symptoms, and incontinence-related quality of life.

**Results:**

The sample size estimate for our trial was 300, but the trial stopped early because of poor recruitment. 74 women participated (40 allocated to TVT Secur, 34 to TVT). At 12 months postoperatively, 27/33(82%) of TVT Secur group were cured, compared with 25/28(89%) of the TVT group (relative risk 0.92, 95% confidence interval 0.75 to 1.13, p = 0.49). Most women reported little or no SUI symptoms (35/37(95%) vs 29/30(97%), >0.999). Quality of life improved significantly from baseline for both groups (IIQ-7 mean change −25 for both groups) but did not differ between groups (p = 0.880).

**Conclusion:**

Our small randomized trial did not find statistically significant differences in outcomes between women allocated to the TVT Secur device versus those allocated to the TVT device for stress urinary incontinence. Despite the discontinuation of TVT Secur in March 2013 for commercial reasons, the importance of our study lies in making evidence available for the many women who had a TVT Secur device implanted and their physicians who may be considering alternative treatments. Our experience illustrates the difficulty of undertaking research on new licensed devices in a rapidly changing surgical specialty, and further highlights the need for research before licensing if surgeons and their patients are to be confident in the effectiveness and safety of new surgical devices.

**Trial registration:**

ClinicalTrials.gov NCT00685217, 22 May 2008.

## Background

In 2006, Ethicon Inc. introduced a new minimally invasive single incision sling device for the surgical treatment of stress urinary incontinence, the Gynecare TVT Secur® (Gynecare, Ethicon Inc., Somerville, MA, USA). For device licensing, the new device was licensed on the basis of evidence of efficacy and safety from a predicate device, the tension-free vaginal tape device (TVT®, Gynecare, Ethicon Inc., Somerville, MA, USA) rather than the new single incision procedure, as is required by device regulations in the USA, Europe and Canada [1]. Gynecare also provided evidence from animal trials of pull-out strength of the novel fixation tips that were part of the new device: these data were not made publicly available.

Urogynecologists have for several years been concerned about the introduction of new devices into clinical practice without new evidence of safety or effectiveness, with the new Gynecare TVT Secur sling device being a catalyst that prompted a number of commentaries [2, 3]. At a meeting of the Western Society for Pelvic Medicine in 2007, the surgeons decided that a trial of TVT Secur was a research priority, given the lack of new clinical evidence to support its adoption into practice. The objective of our randomized trial was to compare the effectiveness of the new TVT Secur sling device to that of the established retropubic TVT device at 12 months postoperatively, with the primary outcome being objective “cure” (urine leakage less than 1 g) measured using a standardized pad test.

Despite the discontinuation of TVT Secur in March 2013 for commercial reasons, the importance of our study lies in making evidence available for the many women who had a TVT Secur device implanted and their physicians who may be considering alternative treatments.

## Methods

Our study was designed as a parallel group randomized trial with follow up of participants at one year postoperatively.

### Settings

The study was conducted by five participating surgeons (three urogynecologists and two general obstetrician/gynecologists) in four centres: two tertiary care academic hospitals in Alberta, Canada, and one academic community hospital and an academic regional hospital in British Columbia, Canada. All participating surgeons had at least 8 years’ experience of incontinence surgery using TVT slings. All undertook preceptored training with TVT Secur, and had carried out at least five of each procedure as lead surgeon before recruiting patients to the trial. The study was approved by the ethics committees of each participating site.

### Participants

Women electing for surgical management of stress urinary incontinence (SUI) were eligible to participate in the trial if they leaked urine with increased abdominal pressure [4, 5], and were suitable for either type of surgery. Women were excluded if they had previous incontinence surgery; required concurrent pelvic organ prolapse surgery; had primary complaint of overactive bladder or incontinence caused only by bladder overflow; intended to have further children; had Alzheimer’s or Parkinson’s disease, progressive neurological disease such as multiple sclerosis, or were immunocompromized; were are unable to understand English or would be unavailable for follow-up. Patients agreeing to join the study provided written informed consent.

### Baseline

Participants were asked to complete a questionnaire including incontinence-related quality of life measures: Urogenital Distress Inventory (UDI-6), a six item measure of urogenital distress, and Incontinence Impact Questionnaire (IIQ-7), a seven-item measure of incontinence effect [6] and a 12-item sexual function questionnaire, the Pelvic Organ Prolapse/Urinary Incontinence Sexual Questionnaire-12, (PISQ-12) [7]. Patient characteristics including obstetrical history and menopausal status were extracted from patient charts.

### Randomisation

Consenting patients were randomly allocated 1:1 to receive a retropubic procedure using either a TVT Secur sling device (“TVT Secur Group”) or the TVT tension-free vaginal tape device (“TVT Group”). The randomisation list was generated by the study analyst (using proc plan procedure in SAS (SAS Institute Inc., Cary, NC, USA) using permuted block randomisation with blocks of varying size (2 – 6) and stratification by surgeon. Neither the surgical team nor the patient knew the next treatment allocation. Randomisation was carried out using an e-mail randomisation service through the University of Calgary Department of Obstetrics and Gynecology data manager. Site research nurses informed the data manager when a random allocation was needed and the patient allocation was notified a few days before surgery to ensure that the appropriate device was available in the operating room: surgeons were unaware of group of allocation until day of surgery. Neither site research nurses nor surgeons were blinded to group of allocation for operational reasons.

### Interventions

Ethicon Gynecare sling devices were used: either the TVT Secur device (using “U” technique, akin to retropubic placement) or the TVT device. All procedures were carried out according to the usual practice of participating surgeons, consistent with the recommendations of the device manufacturer. Preoperative urodynamic testing was used at the clinician’s discretion [5]. Anaesthesia included general, regional or local anaesthesia with sedation, depending on the choice of surgeon, anaesthesiologist and patient. Intraoperative cystoscopy was carried out at the end of the procedure for all patients. Where possible the operations were planned as day procedures, with postoperative home care (depending on usual hospital practice). If necessary for clinical or logistical reasons, women were admitted to the hospital. Surgical details including type of anaesthesia, length of operation, length of time in hospital (hours) and any intra- or post-operative complications (including any difficulty experienced with the procedure) were extracted from patient charts, verified as necessary by surgeon.

If women required re-operation for incontinence during the 12 months following surgery, the appropriate procedure was left to the surgeon’s discretion.

### Outcomes

Outcomes were measured at 12 months following surgery. All participating women were invited for outpatient follow-up. Chart data on outcomes were collected by study research nurses. Women who were unable to attend clinic follow-up were asked to complete questionnaires including subjective outcome. Women were not informed which device they had received.

#### Objective cure (primary outcome)

Objective evidence of SUI was obtained using a standardized pad test at 12 months after surgery. Women undertaking the test had retrograde bladder filling with 300 ml of sterile water and wore pre-weighed pads while they undertook the standardized physical activities of the 1-hour pad test [4]. Women were considered “cured” if the pad weight gain was less than 1 g over the test period, a definition of cure used in other studies of surgery for SUI [8–10].

#### Complications and adverse events

Complications were identified from hospital and routine 6-week follow-up charts. At the 12 month follow-up, a physician carried out an examination of operative wounds and a digital vaginal exam to palpate for tape erosion. Women were also asked to recall any problems they believed they experienced as a result of surgery.

#### Subjective evidence of cure

Subjective cure at 12 months after surgery was defined as either no experience of “lost or leaked urine when you coughed, laughed, sneezed, lifted, exercised, etc.”, or if urine loss has been “a small problem” or “no problem at all” over the past seven days.

#### Incontinence-related quality of life

All women were asked at 12 months to complete UDI-6 and IIQ-7 [6]. Each measure produces a single score of 0 (no distress for UDI-6, no impact for IIQ-7) to 100 (maximum distress or impact). Both measures were developed for incontinence trials, have been independently validated [11–13], are widely used [14] and endorsed by the International Consultation on Incontinence (ICI) [15].

#### Sexual function

At 12 months, women were asked if they had returned to usual sexual activity. Sexual function was measured using PISQ-12, which produces a single score of 0 (poor function) to 48 (excellent function) [7] and is recommended by ICI for measuring sexual function in patients with incontinence [15].

#### Satisfaction with surgical outcome

Women were asked whether the outcome of surgery had met expectations and whether they would recommend the surgery to someone else.

### Sample size

In 2007 when our study was designed, we assumed the cure rate for the TVT Group would be 73% [9], and wished to be able to identify a 10% difference in cure rate for the new TVT Secur device (either worse or improved outcome compared to TVT), a difference the investigators believed would be required to change clinical practice (to support or restrict its introduction). With these assumptions, 80% power, significance level of 0.05, and a drop-out rate of 7% (based on follow-up in our previous randomized trial [8]), we estimated that a sample of 150 patients per group would be required (total 300). Recruitment was estimated to take around a year.

Unfortunately surgeon and centre recruitment was affected by a number of issues, including uncertainty about TVT Secur outcomes, leading to the loss of enthusiasm for the study by individual surgeons and centres that had originally agreed to take part. In the end, recruitment did not start until February 2009 and was closed in October 2010 at which time it was obvious that the trial could not reach its target recruitment. The final recruit consented in October 2010 and had her procedure in March 2011. The decision to close the study early was endorsed by the trial Data and Safety Monitoring Committee.

### Analysis

Analysis was undertaken following the intention-to-treat principle: women were analysed in the surgical group to which they were randomized even if they received another procedure. A single analysis was planned, when all women had completed 12 month follow-up.

Data entry and management were carried out using Access (Microsoft, Redmond, WA), and analyses were carried out using SAS v9.3 (SAS Institute Inc., Cary, NC, USA). A data entry audit was carried out on all fields of a 10% random sample of records. Missing value, range and logic checks identified other data discrepancies. All complications were reviewed by a urogyneacologist (MR). Descriptive statistics (means, standard deviations, proportions) were calculated for baseline data. Primary analysis compared the proportions of patients in the TVT Secur Group versus the TVT Group who demonstrated cure on the 1-hour pad test at 12 months following surgery, using Fisher Exact Test: relative risk (RR) and 95% confidence interval (CI) were also calculated. Analyses of secondary outcomes used similar tests to compare binomial outcomes for the two groups. Differences between groups for time, quality of life and sexual function scores were compared using the Mann–Whitney U-test. Changes in UDI-6, IIQ-7 and PISQ-12 scores were compared between groups using t-tests. Imputation was not used for missing data, and missing values were excluded from all statistical tests and RRs. Results are reported according to the CONSORT Statement for parallel group randomized trials [16].

### Approvals

Ethics approval was received from the research ethics boards responsible for each of the participating hospitals: University of Calgary Conjoint Health Research Ethics Board, reference E-21528, approved 6 May 2008; University of Alberta Health Research Ethics Board, reference 6862, approved 25 May 2008; Interior Health Research Ethics Board, reference 2008–032, approved 26 September 2008; University of British Columbia, reference H08-02281, approved 25 May 2009.

## Results

**Figure 1:**
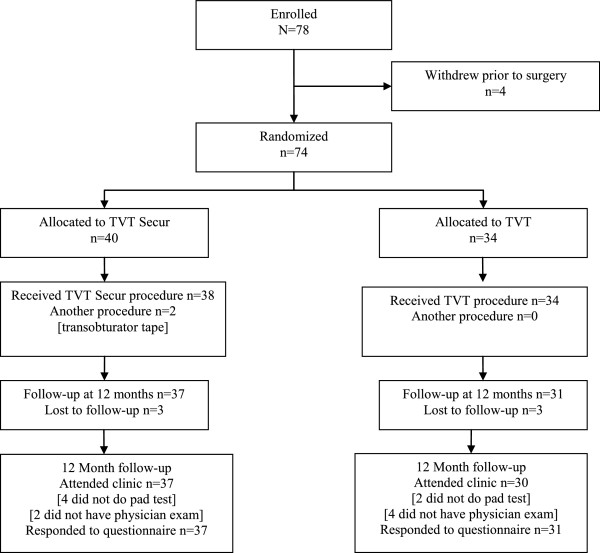
**Flow of patient recruitment and follow-up.**

Figure 1 shows a flow diagram of patient recruitment and follow-up in the study. Four women consented to join the study but eventually chose not to have surgery and were not randomized. Seventy-four women were randomized into the study: academic centres recruited 26 and 27 subjects, the academic community hospital recruited 6 and the academic regional hospital recruited 16. Women had their surgery between February 2009 and March 2011. Forty women were randomly allocated to the TVT Secur Group and 34 to the TVT Group. A total of 68 (92%) were followed up at 12 months.

Baseline data are presented in Table 1. Operative details and hospital stay are described in Table 2. All but two women in the TVT Secur Group had the procedure as allocated: one woman had a transobturator tape device because the random allocation was unavailable in the OR; another woman had the TVT Secur device replaced by a transobturator tape device at the index surgery when the intraoperative cough test found significant SUI (Figure 1). Two subjects (one in each group) had a Novasure endometrial ablation, and one TVT Group patient also had a hysteroscopy, dilatation and curettage at the same time as their SUI procedure. Other operative complications were as follows: in one TVT Secur Group patient, urine continued to leak after the device arms were tightened as much as considered safe but no action was taken to replace the tape; and in another TVT Secur Group patient, after a button hole was formed in the left vagina fornix, the device was repositioned and the button hole repaired. One of the TVT Group patients had a bladder perforation, and one had ≥ 200 ml blood loss. Duration of operation was similar for both groups (TVT Secur Group median 18 minutes versus TVT Group median 21 minutes). Similar numbers of women were admitted to hospital (two (5%) TVT Secur Group versus three (9%) TVT Group patients) mainly for logistic reasons (e.g. time of operation was too late to start home care).

**Table 1:** **Patient characteristics at baseline**

Characteristic	TVT secur group	TVT group
	n = 40	n = 34
**Mean age (years)**	52.4 (SD 12.3)	47.2 (SD 10.8)
**Median BMI**	27.2 (IQR 7.1)	27.8 (IQR 7.8) (1 missing)
**White ethnic group**	39 (98%)	32 (94%)
**Current smoking**	5 (13%)	6 (18%)
**Nulliparous**	2 (5%)	2 (6%)
**No vaginal deliveries**	4 (10%)	4 (12%)
**Constipation**	7 (18%)	11 (32%)
**Postmenopausal**	18 (45%)	11 (32%)
**Currently on hormone replacement treatment**	11 (28%)	5 (15%)
**Questionnaire findings**		
**Median UDI-6 Score**	39 (IQR 25)	39 (IQR 22) (1 missing)
**Median IIQ-7 Score**	33 (IQR 29) (2 missing)	36 (IQR 24)
**Median PISQ-12 Score**	36 (IQR 9) 13 NA, 1 missing	37 (IQR 9) 5 NA
**Stress UI symptoms in past 7 days**		
No, or Yes but no problem or small problem	20 (50%)	17 (50%)
Yes, a big problem	19 (48%)	17 (50%)
Yes, unknown how much problem	1 (3%)	0 (0%)
**Urge UI symptoms in past 7 days**		
No, or Yes but no problem or small problem	32 (80%)	31 (91%)
Yes, a big problem	7 (18%)	3 (9%)
Unknown/Yes, unknown how much problem	1 (3%)	0 (0%)
**Night time awakening to void in past 7 days**		
No, or Yes but no problem or small problem	35 (88%)	33 (97%)
Yes, a big problem	2 (5%)	1 (3%)
Unknown/Yes, unknown how much problem	3 (8%)	0 (0%)

Notes: SD – standard deviation; BMI - Body Mass Index; IQR – inter-quartile range; NA - not applicable.

**Table 2:** **Operative and hospital details**

	TVT secur group	TVT group	Statistical test results
	n = 40	n = 34	
Median operation time (procedure start to closure, mins)	18 (IQR 11)	21 (IQR 12)	M-W p = 0.23
**Type of anaesthesia**			FE p = 0.56
Local only	19 (48%)	16 (47%)	
General only	19 (48%)	18 (53%)	
Spinal with/without local	2 (5%)	0 (0%)	
Complications*			FE p > 0.99
Yes	3 (8%)	2 (6%)	
No	36 (92%)	31 (94%)	
Unknown	1	1	
**Hospital admission**			FE p = 0.66
Not admitted	38 (95%)	31 (91%)	
Planned	2 (5%)	3 (9%)	

Notes: IQR – inter-quartile range; M-W – Mann–Whitney U-test; FE – Fisher Exact Test.

The primary outcome, objective cure at 12 months postoperatively, was measured using a pad test in 33 women in the TVT Secur Group and 28 in the TVT Group (Table 3). Of women in the TVT Secur Group who had a pad test, 27/33 (82%) were “cured”, compared to 25/28 (89%) in the TVT Group (relative risk 0.92, 95% CI 0.75 to 1.13). Other pelvic procedures were reported for 4 (11%) women in the TVT Secur Group, only one of which was linked to the SUI procedure: an excision of the vaginal mesh for an extrusion in the patient whose button hole was repaired. No additional pelvic floor procedures were reported for the TVT Group. At follow-up, other complications during the 12 month postoperative period were reported by three women in the TVT Secur Group, who reported worsening of nocturia, pain in the groin, and development of leakage following the tape excision for an erosion (respectively): one woman in the TVT Group reported an occasional burning sensation under the urethra. On vaginal examination, the majority of women had normal palpation, but the surgical tape was palpable (non-tender) for seven (21%) women in the TVT Secur Group and two (8%) in the TVT Group.

**Table 3:** **12 month follow-up**

	TVT secur n = 37	TVT n = 30	Fisher exact test results*	RR (95% CI)*
**Pad test (primary Outcome)**			p = 0.49	0.92
<1 g	27 (82%)	25 (89%)		(0.75 – 1.13)
≥1 g	6 (19%)	3 (11%)		
Pad test not done	4	2		
**Complications since hospital discharge**			p = 0.13	0.89 (0.79 – 1.00)
Additional pelvic procedures	31 (89%)	26 (100%)		
No	4 (11%)	0 (0%)		
Yes	2	4		
Unknown	1 (2%)	0 (0.0%)		
Surgery for mesh extrusion				
**Vaginal examination**			p = 0.27	0.85
Normal palpation	26 (79%)	24 (92%)		(0.69 - 1.05)
Tape palpable but non-tender	7 (21%)	2 (8%)		
Unknown/not done	4	4		
**Bimanual examination**			p = 0.50	0.94
Normal	32 (94%)	25 (100%)		(0.87 – 1.02)
Abnormal	2 (6%)	0 (0%)		
Unknown/not done	3	5		

Note: *Tests exclude women did not have test or exam, or whose status is unknown.

Questionnaires were completed by 37/40 (93%) women in the TVT Secur Group and 31/34 (91%) in the TVT Group (Table 4). Incontinence symptoms did not differ between groups: subjective cure (no or little problem with stress incontinence symptoms) was reported by women 35 (95%) in the TVT Secur Group, and 29 (97%) in the TVT Group. Quality of life had improved for both groups as reported using UDI-6 and IIQ-7 but did not differ between groups: mean decrease in UDI-6 score of 28 for the TVT Secur Group and 27 for the TVT Group; mean decrease in IIQ-7 score of 25 for both groups. The majority of women reported the surgery met their expectations (26 (70%) versus 28 (90%), p = 0.069), and would recommend the surgery to someone else with similar symptoms (34 (92%) versus 30 (97%), p > 0.99). Sexual activity was reported at baseline and 12 months by 22 women in the TVT Secur Group and 26 in the TVT Group: PISQ-12 scores improved by median 4 points in both groups (t-test p = 0.77).

**Table 4:** **12 month follow-up questionnaire results**

	TVT Secur group n = 37	TVT group n = 31	Statistical results	RR (95% CI)
**Stress UI symptoms in past 7 days**			FE p > 0.99	0.98
No, or Yes but no problem or small problem	35 (95%)	29 (97%)		(0.88 – 1.08)
Yes, a big problem	2 (5%)	1 (3%)		
Unknown/Yes, unknown how much problem	0	1		
**Urge UI symptoms in past 7 days**			FE p = 0.59	1.04
No, or Yes but no problem or small problem	36 (97%)	29 (94%)		(0.93 – 1.16)
Yes, a big problem	1 (3%)	2 (6%)		
**Night time awakening to void in past 7 days**			FE p = 0.50	0.95 (0.88 – 1.02)
No, or Yes but no problem or small problem	35 (95%)	31 (100%)		
Yes, a big problem	2 (5%)	0 (0%)		
**Median UDI-6 score**	11 (IQR 17)	0 (IQR 11)	M-W p = 0.22	n/a
**Mean change from baseline**	−28 (SD 21)	−27 (SD 20)	t-test p = 0.84	n/a
**Median IIQ-7 score**	0 (IQR 14)	0 (IQR 14)	M-W p = 0.71	n/a
**Mean change from baseline**	−25 (SD 27)	−25 (SD 18)	t-test p = 0.88	n/a

Notes: Tests exclude women did not have test or exam, or whose status is unknown.

## Discussion

### Main findings

Our trial is the first randomized trial to compare the outcomes of TVT Secur using the U-method with outcomes following the established TVT in women with stress urinary incontinence without other pelvic floor surgical procedures, and no additional unpublished trials of this design are registered. The two tape devices are designed to place the tape in the same position in relation to the urethra, and therefore our study provides a direct comparison between the devices. At 12 months postoperatively we did not find statistically significant differences in outcome between groups for cure defined as pad test leakage of <1 g (82% TVT Secur versus 89% TVT), or subjective cure defined as no or small problem caused by leakage in the past week (95% TVT Secur versus 97% TVT). Nor did we find differences between groups for incontinence-related quality of life or adverse effects following surgery.

### Comparison of the findings with published literature

Only one randomized trial has been published to date directly describing the outcome of TVT Secur U technique to that following TVT [17]. This large trial, by Barber and colleagues, compared outcomes a year postoperatively, differing from our trial in including women with pelvic organ prolapse or other pelvic floor problems. Among the 263 subjects randomized, 65% had concomitant surgery (hysterectomy, pelvic organ prolapse surgery or colpolcleisis) [17]. Barber and colleagues’ trial did not include an objective outcome measure, rather reporting on subjective cure (a composite of subjective report of Incontinence Severity Impact [18] and absence of other incontinence treatments) at 12 months. The study found that 57% of women were cured among the TVT Secur group, versus 61% of the TVT group (TVT Secur was not found inferior to TVT), but significantly more patients in the TVT Secur group reported severe symptoms (16% versus 5%, p = 0.025). The lower success rates in Barber’s study (compared to our cure rates of 82% and 89%) is likely as a result of the difference in outcome definition compared to ours, and may also have been influenced by the concomitant surgery. In Barber’s study, over 90% in each group would choose the same procedure again, and women in both groups demonstrated improvements from baseline UDI-6 and IIQ-7 scores that were similar to the changes experienced by the women in our study.

One other published randomized trial compared TVT Secur (using the hammock (H) technique that places the tape in a position replicating an obturator tape) to TVT, recruiting 125 women, less than half of the planned 280 patients [19, 20]. Recruitment was stopped early because of a significantly lower cure rate at 2 months, and concern about three severe adverse events in the TVT Secur group (a tape erosion into the urethra, a tape placed inside the bladder, and bleeding from the corona mortis) [19]. Follow-up at one year after surgery found that TVT Secur produced significantly poorer outcomes than TVT, with fewer women having no leakage on cough test (71% TVT Secur (H) versus 94% TVT, p = 0.01), no pad test leakage (58% TVT Secur (H) versus 94% TVT, p = 0.05), and subjective cure (80% TVT Secur (H) versus 98% TVT, p = 0.03) [20]. The authors suggested that despite careful training in the TVT Secur H technique, their adverse findings for TVT Secur might have been the result of the TVT Secur being more clinician-dependent and less “forgiving”, requiring extremely careful placement of the “one chance only” device [19]. Another randomized trial compared TVT Secur outcome to TVT and TVT-O (obturator tape), finding TVT Secur was least effective at one year after surgery (subjective cure 68% TVT Secur, 94% TVT, 92% TVT-O, p = 0.005) [21]. Several other randomized trials reported on outcome of TVT Secur (U or H approach) compared to transobturator tape devices, finding lower objective and/or subjective cure rates than transobturator tape procedures [22–26] or that TVT Secur was not inferior to transobturator tape procedures [27–30]. As a result of their findings, trial authors were generally critical of TVT Secur.

Like Barber et al’s research [17], our study found little difference between outcomes for TVT Secur and TVT. We have speculated about possible reasons for the positive outcomes in our study compared to other studies in which TVT Secur produced lower effectiveness compared to more traditional slings. One potential reason might be that the participating surgeons opted to undertake two TVT Secur training sessions (supported by Gynecare). This was prompted by circulating rumours in 2008, before published evidence was available, that TVT Secur required a precise technique. Our second preceptor stressed the need for “physiologic tensioning” using intraoperative cough or Crede testing to fine tune positioning of the mesh, and offered a technique to prevent mesh movement as inserters were removed. These fine details were in stark contrast to the simpler established retropubic tension-free vaginal tape procedure. The second round of preceptored training led to delay in the start of our trial, and may have raised surgeons’ concerns about TVT Secur, contributing to poor trial recruitment by collaborating surgeons.

Two rigorous systematic reviews published recently investigated outcomes following single-incision mini-slings: one was a systematic review with meta analysis [31] and one a Cochrane review [32]. Both of these reviews commented specifically on outcomes following TVT Secur versus more traditional TVT and transobturator tapes. Both came to similar conclusions: that TVT Secur was not as effective as the more traditional tape devices and leads to more adverse effects (such as tape erosions and reoperations). Both of the reviews attributed the 2013 decision to withdraw TVT Secur from the market to these findings. The reviews found that there was too little evidence about the outcomes of other types of single incision mini-slings to comment confidently on the outcomes of those devices. Adverse outcomes may yet be attributed to those other mini-slings.

### Strengths and limitations

The main strengths of our study are that it compared outcomes for women a year following TVT Secur and TVT, without concomitant pelvic floor surgery. We were able to obtain outcome data for 92% of women recruited, indicating the high level of commitment of the participating centres. The study was carried out in a variety of hospital settings to increase the generalizability of the findings. A further strength was that most of the outcomes were patient-reported by subjects who were not made aware which device had been implanted, and whose potential preferences or biases for one or other device would not affect their perception of outcome. The primary outcome, urine leakage measured during a standardized pad test was undertaken by unblinded but independent outcome assessors, research nurses who were not involved in patient care.

The principal limitation of our study was that it stopped early because of poor recruitment, with only 74 patients randomized, rather than the planned 300. Although recruitment to surgical trials is well known to be difficult, we were surprised that this affected our study, given the 2007 enthusiasm of the Western Society for Pelvic Medicine surgeons. Two surgeons who initially expressed interest eventually chose to carry out their own local trials, others felt their centres had too few eligible patients or did not have research support locally. Delays caused in seeking and obtaining funding for our trial, and providing adequate surgical training - at a time when other new devices became available - meant that enthusiasm for TVT Secur was insufficient to be able to recruit additional centres outside the Western Society for Pelvic Medicine. Surgeons wishing to undertake future studies of new devices should learn from our experience, although identifying a device that will prove to be the leading brand will always be a challenge.

With so few patients in our study, there is a risk that we were unable to identify a real difference between outcomes for the two groups: lack of statistically significant differences in outcome in our study could be as a result of type II error. In a study of this size, only large differences in outcome between groups would be found to be statistically significant. None the less, the findings from our multicentre trial add to the available evidence of effectiveness of TVT Secur compared to TVT that will be of interest to physicians who used TVT Secur and women who had one of these devices implanted.

## Conclusion

The goals of introducing new minimally invasive devices are to improve patient outcomes and ease of use for surgeons, often by simplifying procedures while reducing operating room time. Such goals are laudable, but must be supported by rigorous evidence. Our small randomized trial did not find statistically significant differences in outcomes (cure, adverse events, or quality of life) between women allocated to having a TVT Secur versus those allocated to having an established TVT procedure for stress urinary incontinence. Our trial highlights the need for surgeons to seek adequate training and independent evidence of effectiveness and safety before they adopt new devices into their clinical practice. Our experience also illustrates the difficulty of undertaking research on new licensed devices in a rapidly changing surgical specialty, and further highlights the need for research before licensing if surgeons and their patients are to be confident in the effectiveness and safety of new surgical devices. Our study reiterates the wisdom of the recommendations of the 2008 NICE guidelines for single incision sub-urethral slings, highlighting the need to introduce these types of device only in the context of research studies or national registers [33].

## Abbreviations

H: Hammock technique for TVT Secur placement, akin to obturator tape placement
ICI: International consultation on incontinence
IIQ-7: 7 item incontinence impact questionnaire
PISQ-12: 12 item pelvic organ prolapse/urinary incontinence sexual questionnaire
SUI: Stress urinary incontinence
TVT: Tension-free vaginal tape
U: Technique for TVT secur placement, akin to retropubic tape placement
UDI-6: 6 item urogenital distress inventory.
